# Cardiovascular and Respiratory Effect of Yogic Slow Breathing in the Yoga Beginner: What Is the Best Approach?

**DOI:** 10.1155/2013/743504

**Published:** 2013-04-23

**Authors:** Heather Mason, Matteo Vandoni, Giacomo deBarbieri, Erwan Codrons, Veena Ugargol, Luciano Bernardi

**Affiliations:** ^1^Department of Neuroscience, Roehampton University, London, UK; ^2^Department of Public Health and Neuroscience, University of Pavia, Pavia, Italy; ^3^Department of Internal Medicine, University of Pavia and IRCCS San Matteo, Pavia, Italy; ^4^Department of Psychology, The Open University, London, UK

## Abstract

Slow breathing increases cardiac-vagal baroreflex sensitivity (BRS), improves oxygen saturation, lowers blood pressure, and reduces anxiety. Within the yoga tradition slow breathing is often paired with a contraction of the glottis muscles. This resistance breath “ujjayi” is performed at various rates and ratios of inspiration/expiration. To test whether ujjayi had additional positive effects to slow breathing, we compared BRS and ventilatory control under different breathing patterns (equal/unequal inspiration/expiration at 6 breath/min, with/without ujjayi), in 17 yoga-naive young healthy participants. BRS increased with slow breathing techniques with or without expiratory ujjayi (*P* < 0.05 or higher) except with inspiratory + expiratory ujjayi. The maximal increase in BRS and decrease in blood pressure were found in slow breathing with equal inspiration and expiration. This corresponded with a significant improvement in oxygen saturation without increase in heart rate and ventilation. Ujjayi showed similar increase in oxygen saturation but slightly lesser improvement in baroreflex sensitivity with no change in blood pressure. The slow breathing with equal inspiration and expiration seems the best technique for improving baroreflex sensitivity in yoga-naive subjects. The effects of ujjayi seems dependent on increased intrathoracic pressure that requires greater effort than normal slow breathing.

## 1. Introduction

Respiratory research documents that reduced breathing rate, hovering around 5-6 breaths per minute in the average adult, can increase vagal activation leading to reduction in sympathetic activation, increased cardiac-vagal baroreflex sensitivity (BRS), and increased parasympathetic activation all of which correlated with mental and physical health [1–4]. BRS is a measure of the heart's capacity to efficiently alter and regulate blood pressure in accordance with the requirements of a given situation. A high degree of BRS is thus a good marker of cardiac health [5].

The slow breathing-induced increase in BRS could be due to the increased tidal volume that stimulates the Hering-Breuer reflex, an inhibitory reflex triggered by stretch receptors in the lungs that feed to the vagus [6]. In addition, the slow breathing increases the oxygen absorption that follows greater tidal volume (*V*_*t*_), as a result of reduction in the effects of anatomical and physiological dead space [7, 8]. This might in turn produce another positive effect, that is, a reduction in the need of breathing. Indeed, a reduction in chemoreflex sensitivity and, via their reciprocal relationships, an increase in BRS, have been documented with slow breathing [9–13]. 

Ujjayi resistance breathing, a breathing practice taught by the yogic tradition, reduces airflow, and during expiration it increases the intrathoracic pressure due to a slight contraction of the glottis muscles, potentially resulting in intensified vagal activity [14–16]. The increase in expiratory intra-thoracic pressure should also enhance oxygen absorption above what is found in slow breathing, potentially elevating blood pressure levels more than with slow breathing alone and inducing greater BRS [17]. Lastly, ujjayi breathing facilitates greater control over airflow and, therefore, breath rate [14]. Consequently, ujjayi may be a more effective method than slow breathing on its own as a means to achieve five breaths per minute for the average person who tends to breathe at 12–18 breaths per minute. On the other hand, the additional effort to exhale, induced by the increased intra-thoracic pressure, might stimulate sympathetic activity, partially reducing the advantages of this technique. Accordingly, we tested whether ujjayi breath would improve oxygen saturation and BRS, more than slow breathing alone. 

## 2. Materials and Methods

### 2.1. Subjects

The protocol of this study was approved by the Ethics Committee of the University of Pavia, Italy, and all participants gave informed consent to participate in this study. Using a within-participants design, 17 young healthy participants were recruited by word of mouth through university students and staff. Participants provided information pertaining to their general level of fitness, level of sport undertaken (including specialties that lead to practices similar to yoga like diving and martial arts), smoking habits, and average alcohol consumption. These and the anthropometric characteristics of the subjects are shown in Table 1.

### 2.2. Protocol

The electrocardiogram was recorded using a bipolar precordial lead. Continuous blood pressure was monitored with a digital plethysmograph (Portapres, FMS Medical Systems, Amsterdam, The Netherlands) from the middle finger of the right arm held at heart level. Two respiratory signals were obtained by inductive plethysmography, from belts positioned around the chest and the abdomen. Pulse oximetry and expired carbon dioxide partial pressure (Cosmo, Novametrix, Wallingford, CT, USA) were also obtained. 

Pretesting, participants spent approximately 10 minutes learning how to engage ujjayi breath with a qualified yoga teacher. They were then connected to the measuring devices ready for testing. The testing phase comprised 7 conditions distinguished by breathing rate and inclusion or not of ujjayi breath. Although the effects described for ujjayi should essentially occur during expiration (as normally practiced), we also included an evaluation of ujjayi during both inspiration and expiration, as suggested by some yoga teachers. We decided to perform the ujjayi without the addition of so-called “bandhas” (i.e., contractions at the level of perineum or abdomen or tucking the chin close to the chest), since in yoga naive participants these additional movements could be difficult to perform without practice. This is also in agreement with many yoga schools, which do not necessarily associate the bandhas with ujjayi. The recordings were made in the supine position during 3 minutes spontaneous breathing, during 2 minutes controlled breathing at a frequency similar to normal spontaneous breathing (15 breaths/minute), and during 2 minute periods of slow deep breathing at the rate of 6 cycles/minute with either equal or unequal inspiration/expiration ratio and with or without ujjayi (Table 2 reports the methodology for the different recordings). 

All recordings were performed in random order, except baseline that was always performed first. Each recording was separated by the previous one by 2 minutes.

All signals were simultaneously acquired on a personal computer with an analog-to-digital converter with a 12-bit resolution at a sampling rate of 400 Hz on a Macintosh computer using a special software written in our laboratory.

### 2.3. Assessment of BRS

From the original data the time series of RR interval (from each of 2 consecutive *R* waves of the electrocardiogram) and systolic blood pressure (SBP) were obtained. Previous studies have shown a poor correlation between different indices of BRS, while on the other hand, no method has shown clear superior performance over the other [18]. Accordingly, we computed a set of 7 different tests and used their average [19]. BRS was determined from spontaneous fluctuations in the RR interval and SBP during the spontaneous, 15/min and 6/min recordings using the positive and negative sequence methods [20], the alpha coefficient in the low and high frequency bands and its average [21], and the transfer function technique [22]. In the sequence methods, the BRS was estimated by identifying spontaneously occurring sequences of 3 or more consecutive heartbeats in which both the SBP and the subsequent RR intervals changed in the same direction. The minimum criteria for change were 1 mm Hg for SBP and 5 ms for the RR intervals. For identified positive and negative sequences with a correlation coefficient between the RR intervals and the SBP exceeding 0.85, the regression slopes (the slope of the regression line between SBP and RR intervals) were calculated, and the average was taken as a measure of BRS positive and negative slopes, respectively. The other 4 BRS methods were calculated by autoregressive uni- and bivariate spectral analysis. The alpha coefficient was calculated as the square root of the ratio of the powers of RR intervals and SBP in the low frequency range (0.04–0.15 Hz) and in the respiratory (0.15–0.40 Hz) high frequency range when coherence was greater than 0.5, and the phase difference between SBP and RR intervals was negative. In the transfer function method BRS was calculated as the average value of SBP-RR cross-spectrum divided by the SBP spectrum in the low frequency range (0.04–0.15 Hz), when coherence exceeded 0.5. The last method was obtained by the standard deviation of RR interval divided by the standard deviation of SBP after a high-pass filtering at 0.050 Hz corner frequency, 6 dB/octave attenuation, as recently proposed and validated [19].

### 2.4. Analysis of Respiration

The signals from the inductive plethysmographic belt signals were analyzed by an interactive program to identify for each breath the positive and negative respiratory peaks, together with the respiratory period. The sum of the signals obtained by the 2 belts was taken as a relative index of tidal volume. Additionally, the same program automatically identified the end-expiratory (end-tidal) value in the carbon dioxide signal. Using the inductive belt data a semiquantitative intrasubject analysis of ventilation could be obtained, by comparing the relative changes in *V*_*t*_ and minute ventilation (*V*_*E*_) induced by oxygen inhalation or different breathing patterns. Although the device used for the present study does not allow to obtain *V*_*t*_ and minute ventilation in absolute values (mL and L/min, resp.), we took advantage of the strong linear relationship between *V*_*t*_ and the inductive belt signals [23], thus allowing us to obtain ventilation in relative units. Great care was taken to ascertain that the belts would not be displaced during the experiment. The limitation of the semiquantitative analysis is compensated by the lack of interference with the spontaneous breathing, that typically occurs with mouthpieces [24]. We therefore set the minute ventilation obtained during spontaneous breathing (our baseline) as 100% in each subject and calculated the minute *V*_*E*_ or *V*_*t*_ in % changes from that value for each recording [25].

### 2.5. Estimates of Chemoreflex Sensitivity

Although it was practically impossible to practice this type of respiration during a typical chemoreflex testing (requiring a rebreathing circuit and a mouthpiece or a face mask) [11], we could still use a previously validated simpler and approximative index of chemoreflex sensitivity, based on the ratio of tidal volume to inspiratory time (*V*_*t*_/*T*_*i*_) [26]. Because we evaluated the *V*_*t*_ only in relative units, we used the same normalisation procedure used for ventilation data and thus expressed the values obtained in each recording as variations from the baseline (set to 100% in each subject). 

### 2.6. Statistical Analysis

Data are presented as mean ± standard error of the mean (SEM). Statistical differences between baseline and the different interventions (6/min versus 15/min controlled breathings) were tested by analysis of variance for repeated measures (ANOVA) [27]. Sheffe' test was used to test for significances between different breathing techniques. Statistical significance was defined as a value ≤0.05. All comparisons were done with respect to spontaneous breathing and also with controlled breathing at a frequency similar to the spontaneous (15 breath/min), to identify the effect of controlling respiration per se.

## 3. Results

**Figure 1:**
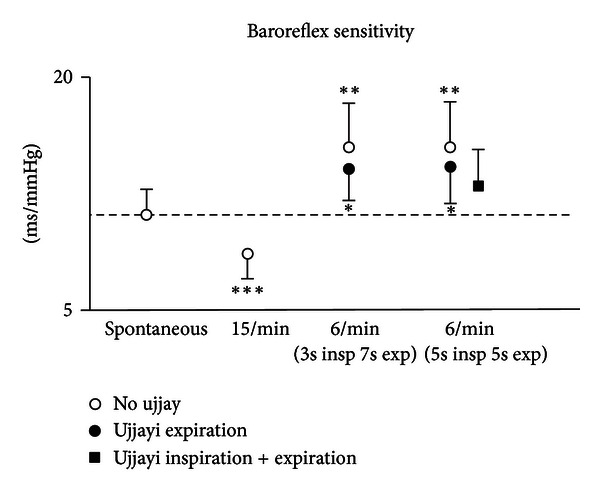
Effect of breathing techniques on BRS values (*P < 0.05; **P < 0.01; ***P < 0.001, versus spontaneous breathing).

**Figure 2:**
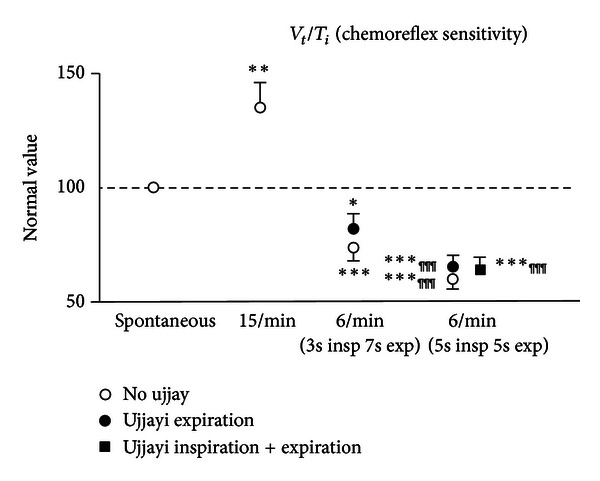
Effect of breathing techniques on estimated chemoreflex sensitivity values (*P < 0.05; **P < 0.01; ***P < 0.001, versus spontaneous breathing; ^¶¶¶^ *P* < 0.001, versus 6/min (3 s inspiration + 7 s expiration)).

Complete results are shown in Table 3 and Figures 1 and 2. Overall, data were consistent, and we did not find significant differences between male and female participants.

### 3.1. BRS (Figure 1)

In comparison to spontaneous breathing, fast breathing led to a reduction in BRS, whilst all slow breathing (with or without ujjayi breathing) increased BRS. This increase was seen in both the symmetrical (5 second inspiration and expiration) and asymmetrical (3 second inspiration and 7 second expiration) slow breathing conditions. Engaging ujjayi breathing on the exhalation had the effect of reducing the increase in BRS of slow breathing alone, and this was further reduced with ujjayi on the inspiration and expiration (which was not significantly higher than baseline). These differences were even more pronounced with respect to controlled breathing at 15 breath/minute, which also showed highly significant differences with respect to spontaneous breathing, but in the opposite direction. 

### 3.2. Oxygen Saturation, Carbon Dioxide, and Ventilation

Both slow breathing and 15 breath/minute controlled breathing increased oxygen saturation as compared to baseline. When slow breathing was done in conjunction with ujjayi breathing, oxygen saturation further increased, though only slightly. Overall, however, this was a highly significant change given that baseline oxygen saturation was already high approximately 98.3% (Table 3). The increase in oxygen saturation during slow breathing was lower than that observed during fast breathing. However, with 15 breath/minute controlled breathing the increase in oxygen saturation occurred with a large relative increase in *V*_*E*_ and a marked drop in end-tidal carbon dioxide. Conversely, with slow breathing, the increase in oxygen saturation occurred with only a moderate increase in *V*_*E*_ and drop in carbon dioxide. Actually, the slow breathing with equal inspiration and expiration time showed a similar increase in oxygen saturation without a significant increase in *V*_*E*_ as compared to baseline.

### 3.3. Heart Rate and Blood Pressure

Except slow breathing with equal inspiration and expiration time, all slow breathing reduced RR interval (increased heart rate). Ujjayi breathing increased heart rate more in comparison to slow breathing alone. Slow breathing reduced both SBP and diastolic blood pressures, particularly when performed with equal inspiration and expiration time. Ujjayi reduced the drop in blood pressure induced by simple slow breathing (Table 3).

### 3.4. Estimates of Chemoreflex Sensitivity (Figure 2)

The ratio *V*_*t*_/*T*_*i*_, a simplified marker of chemoreflex sensitivity, increased with 15 breath/minute controlled breathing and lowered with slow breathing, with a pattern clearly opposite to that of BRS. Accordingly, the largest decrease in *V*_*t*_/*T*_*i*_ was observed during slow breathing with equal inspiration and expiration time (Figure 2).

As slow breathing showed in general opposite results than 15 breath/minute controlled breathing (Table 3 and Figures 1 and 2), all observed changes were more significant when slow breathing with and without ujjayi was compared to fast breathing for all the variables considered.

## 4. Discussion

The present study found that, in nearly all forms of slow breathing performed in yogic breathing naive participants, there were increased BRS (only slow breathing with ujjayi during inspiration and expiration did not result statistically significant) and oxygen saturation, with reduced blood pressures and chemoreflex sensitivity. The greatest improvement was found in slow breathing without ujjayi, while breathing controlled at a rate of 15/min caused a drop in BRS. In all forms of slow breathing there was a statistically significant increase in oxygen saturation from the mean baseline of 98.3%, confirming the relationship between high levels of oxygen absorption and BRS. However, ujjayi breath showed the greatest saturation (albeit only 0.1–.0.2% percent greater), but it did not correspond to the greatest improvement in BRS, likely due to the increased respiratory effort (as seen by the increased heart rate). The increase in BRS was mirrored by a reciprocal drop in chemoreflex estimate. No significant difference was found between asymmetrical versus symmetrical breathing at 6 breaths per minute. These results show that simple slow breathing with equal inspiration/expiration is the best compromise to obtain positive cardiorespiratory effects in yoga naive subjects.

### 4.1. Oxygen Absorption and BRS

In this study, we show that slow breathing and increased oxygen absorption lead to enhanced BRS. This might result from several possible factors, all interrelated. In theory, the increase in arterial oxygen partial pressure increases blood pressure, which in turn could stimulate the baroreceptors and improve the BRS gain. This was recently observed in healthy [28] and diabetic subjects [25]. The seemingly small extent of the increase in oxygen saturation should not be overlooked. In fact, the haemoglobin dissociation curves states that at higher saturation values small changes reflex large changes in the partial pressure of oxygen. 

Because the oxygen tension (and not oxygen saturation) is the chemoreflex input signal, this explains why in a previous study the administration of oxygen in normoxia induced a significant increase in BRS and parasympathetic activity despite a small increase in oxygen saturation [25]. Thus, the increased oxygen absorption may inhibit the chemoreflex and, by this reciprocal relationship [9, 10], increase BRS. Bernardi et al. (2001) demonstrated that slow breathing reduced chemoreflex sensitivity to both hypoxia and hypercapnia, in part attributing this to an inverse relationship with BRS [11]. It is well understood that the chemoreflex is a mechanism to stabilize blood pH by increasing ventilation. Possibly, the increase in oxygen in ujjayi and slow breathing may inhibit the chemoreflex necessarily stimulating greater BRS. Our findings of the reciprocal BRS and chemoreflex changes are in full support of this concept, within the limitations of the chemoreflex method adopted. Secondly, slow breathing with its increased *V*_*t*_ might induce changes in venous return, altering stroke volume, and enhancing phasic changes in systolic blood pressure, synchronous with breathing, that may in turn enhance BRS [11, 29]. Lastly, a central effect of slow breathing with a direct stimulation of parasympathetic activity could not be excluded. These last factors might explain why in this study the increase in oxygen induced by the slow breathing was associated with a reduction in blood pressures, suggesting some important differences with the simple administration of oxygen.

### 4.2. Ujjayi Breath and BRS

Although ujjayi breath showed the greatest increase in oxygen saturation, it did not coincide with the greatest improvement in BRS when done just on the expiration. We believe that this phenomenon is connected to the necessary effort to perform this type of breath. In agreement with this idea, we found that RR interval dropped by effect of ujjayi with respect to slow breathing alone. Accordingly, the improvement in oxygenation induced by ujjayi could have been counteracted by the increased effort and reduced the effect of parasympathetic stimulation induced by slow breathing alone. When done also on inspiration the increase in BRS was not significantly improved with respect to baseline. In this case, the effects could have mimicked a Mueller manoeuvre. The Mueller manoeuvre is known to strain the heart and would potentially override the parasympathetic effects found in slow breathing [30–32]. Tracing potential neural correlates of ujjayi breath it was suggested that in animals ujjayi-like inspiration is found in conditions of threat and may serve to promote vigilance, thereby mitigating the effects of increased BRS [14]. 

### 4.3. Asymmetrical versus Symmetrical Breathing

We did not find any significant difference between asymmetrical and symmetrical breathing during slow breathing. We suggest that most of these results could be due to the prolonged expiratory time (in fact the 3-second inspiratory time of the asymmetrical breathing was very close to the spontaneous breathing). In the yoga tradition several degrees of asymmetries were adopted. While some of these could have specific effects (and could be matter for further investigations), our results suggest that an expiratory time of at least 5 seconds was sufficient to elicit most of the results observed. 

### 4.4. Limitations

In this study, due to the need of avoiding a mouthpiece, we used a simplified, though validated, technique to assess the chemoreflex sensitivity, the *V*_*t*_/*T*_*i*_ ratio [26]. While using this approach we did impose one term of the ratio by fixing the *T*_*i*_, and we nevertheless leaved *V*_*t*_ free to change. If the manoeuvre was not altering the chemoreflex, then we would have expected a change in *V*_*t*_, such that the ratio would stabilise to the same value as the one at baseline. Since this did not happen, it is likely that this ratio was indeed reflecting some change in the chemoreflex. Additionally, these findings are in full agreement with previous studies [11, 33] in which it was shown by us and others that the slow breathing markedly reduced the chemoreflex sensitivity in yoga naive subjects and also in yoga trainees during spontaneous breathing. Being obtained in yoga naive young participants, it is not obvious that our findings could be exactly replicated in older patients or in patients with long-term practice in yoga. It is logical to expect that habitude in practicing breathing exercise will allow to perform them with less effort and thus with perhaps better results (e.g., lower changes in heart rate), particularly during ujjayi. However, to our best knowledge such information is still lacking, and then it should be tested in future investigations. In this study, we observed the effects of these breathing techniques during their application only. The long-term effects of yoga practice could be of high interest, but the specific contribution of each breathing technique cannot be easily identified, as in general yoga trainees use a variety of many different breathing techniques in addition to postures and mediation. However, the same directional changes have been confirmed both for yoga practice [33–35] and also for the specific effects of slow breathing alone [7].

## 5. Conclusions

Based on our findings, slow breathing with similar inspiration and expiration times appears the most effective and simple way to heighten the BRS and improve oxygenation in normoxia. Ujjayi breath demonstrates limited added benefit over slow breathing done at 6/min in normoxia; however, the effects could be more pronounced in hypoxia, and this could be matter for future investigations. As we did not find a significant difference in symmetrical versus asymmetrical breathing, it is suggested that practitioners can engage in a ratio that is personally comfortable and achieve the same BRS benefit. These findings might be relevant for selecting the optimal strategy to train patients undergoing yoga-based rehabilitation programs, as previous studies have shown that patients with different pathologic conditions (such as heart failure, hypertension, and COPD) may benefit from practice in these slow breathing [2–4, 7], while no contraindications to date have been reported. 

## Figures and Tables

**Table 1:** Characteristics of the participants (mean ± SEM).

Number	17
Sex (men/women)	8/9
Age (years)	27.2 ± 1.1
Weight (kg)	63.4 ± 3.3
Height (m)	1.72 ± 2.5
Body mass index (kg/m^2^)	21.1 ± 0.6
Training sessions frequency per week	2.6 ± 0.3
Energy expenditure per week (mets)	14.6 ± 2.1
Smokers	0.0 ± 0.0
Alcohol (glasses/week)	2.2 ± 0.5
Diving and martial arts practitioners	0.0 ± 0.0

**Table 2:** Conditions tested.

Breath rate	Ujjayi/no ujjayi
(1) Spontaneous—baseline measure	No ujjayi
(2) Fast breathing—15 per minute, 2 second inspiration and expiration	No ujjayi
(3) Slow breathing—6 per minute, 5 second inspiration and expiration	No ujjayi
(4) Slow breathing—6 per minute, 5 second inspiration and expiration	Ujjayi
(5) Slow breathing—6 per minute, 5 second inspiration and expiration	Ujjayi on exhalation only
(6) Slow breathing—6 per minute, 3 second inspiration/7 second expiration	No ujjayi
(7) Slow breathing—6 per minute, 3 second inspiration/7 second expiration	Ujjayi on exhalation only

**Table 3:** Effects respiratory patterns on cardiorespiratory variables.

Variable	Spontaneous	15/min	6/min (3 s inspiration + 7 s expiration)		6/min (5 s inspiration + 5 s expiration)		
	No ujjayi	No ujjayi	No ujjayi	Ujjayi exp	No ujjayi	Ujjayi exp	Ujjayi ins + exp
RR interval (ms)	830 ± 32	769 ± 26***	802 ± 25*	789 ± 24*	832 ± 26^¶¶¶^	809 ± 25^##^	797 ± 20^∗##^
Systolic blood pressure (mmHg)	121.84 ± 5.67	113.96 ± 5.67*	117.91 ± 5.21	120.06 ± 4.58	111.34 ± 4.91^∗∗∗¶¶^	118.46 ± 4.77^#^	116.25 ± 4.97^#^
Diastolic blood pressure (mmHg)	54.78 ± 3.69	51.93 ± 2.88*	53.99 ± 3.14	55.77 ± 3.01	55.14 ± 3.23*	54.55 ± 3.29^#^	54.28 ± 2.91^#^
Oxygen saturation (%)	98.37 ± 0.17	99.19 ± 0.17***	98.90 ± 0.18^##^	98.91 ± 0.14**	98.90 ± 0.19*	98.95 ± 0.17**	98.88 ± 0.18*
End-tidal carbon dioxide (mmHg)	36.26 ± 1.27	26.11 ± 0.98***	31.14 ± 1.13**	30.23 ± 0.97***	30.24 ± 0.68***	29.76 ± 1.22***	29.69 ± 0.90***
*V*_*t*_ (normalized to spontaneous breathing)	100.00 ± 0.00	152.82 ± 14.33**	318.40 ± 28.48***	368.48 ± 38.32^∗∗∗#^	249.12 ± 24.39^∗∗∗¶¶¶^	318.88 ± 34.01^∗∗∗ ### ¶¶^	293.91 ± 30.56^∗∗∗ #¶¶^
*V*_*E*_ (normalized to spontaneous breathing)	100.00 ± 0.00	190.88 ± 17.02***	158.81 ± 11.82***	183.35 ± 15.33***	125.12 ± 9.96^¶¶¶^	160.69 ± 14.88^∗∗∗ ### ¶¶^	149.77 ± 14.71^∗∗ # ¶¶^

*P < 0.05; **P < 0.01; ***P < 0.001, versus spontaneous breathing.

^#^ *P* < 0.05; ^##^ *P* < 0.01; ^###^ *P* < 0.001, versus no ujjayi.

^¶^ *P* < 0.05; ^¶¶^ *P* < 0.01; ^¶¶¶^ *P* < 0.001, versus 6/min (3 s inspiration + 7 s expiration).

## References

[1] Brown RP, Gerbarg PL (2005). Sudarshan Kriya Yogic breathing in the treatment of stress, anxiety, and depression: Part II—clinical applications and guidelines. *Journal of Alternative and Complementary Medicine*.

[2] Bernardi L, Porta C, Spicuzza L (2002). Slow breathing increases arterial baroreflex sensitivity in patients with chronic heart failure. *Circulation*.

[3] Joseph CN, Porta C, Casucci G (2005). Slow breathing improves arterial baroreflex sensitivity and decreases blood pressure in essential hypertension. *Hypertension*.

[4] Raupach T, Bahr F, Herrmann P (2008). Slow breathing reduces sympathoexcitation in COPD. *The European Respiratory Journal*.

[5] De Ferrari GM, Sanzo A, Bertoletti A, Specchia G, Vanoli E, Schwartz PJ (2007). Baroreflex sensitivity predicts long-term cardiovascular mortality after myocardial infarction even in patients with preserved left ventricular function. *Journal of the American College of Cardiology*.

[6] Wang H, Zhang H, Song G, Poon CS (2008). Modulation of hering-breuer reflex by ventrolateral pons. *Advances in Experimental Medicine and Biology*.

[7] Bernardi L, Spadacini G, Bellwon J, Hajric R, Roskamm H, Frey AW (1998). Effect of breathing rate on oxygen saturation and exercise performance in chronic heart failure. *The Lancet*.

[8] Oneda B, Ortega KC, Gusmão JL (2010). Sympathetic nerve activity is decreased during device-guided slow breathing. *Hypertension Research*.

[9] Narkiewicz K, van de Borne P, Montano N, Hering D, Kara T, Somers VK (2006). Sympathetic neural outflow and chemoreflex sensitivity are related to spontaneous breathing rate in normal men. *Hypertension*.

[10] Somers VK, Mark AL, Abboud FM (1991). Interaction of baroreceptor and chemoreceptor reflex control of sympathetic nerve activity in normal humans. *Journal of Clinical Investigation*.

[11] Bernardi L, Gabutti A, Porta C, Spicuzza L (2001). Slow breathing reduces chemoreflex response to hypoxia and hypercapnia, and increases baroreflex sensitivity. *Journal of Hypertension*.

[12] Steinback CD, Salzer D, Medeiros PJ, Kowalchuk J, Shoemaker JK (2009). Hypercapnic vs. hypoxic control of cardiovascular, cardiovagal, and sympathetic function. *The American Journal of Physiology*.

[13] Francis DP, Davies LC, Willson K, Ponikowski P, Coats AJS, Piepoli M (2000). Very-low-frequency oscillations in heart rate and blood pressure in periodic breathing: Role of the cardiovascular limb of the hypoxic chemoreflex. *Clinical Science*.

[14] Brown RP, Gerbarg PL (2005). Sudarshan Kriya yogic breathing in the treatment of stress, anxiety, and depression: Part I—neurophysiologic model. *Journal of Alternative and Complementary Medicine*.

[15] Jovanov E On spectral analysis of heart rate variability during very slow yogic breathing.

[16] Ruan T, Ho CY, Kou YR (2003). Afferent vagal pathways mediating respiratory reflexes evoked by ROS in the lungs of anesthetized rats. *Journal of Applied Physiology*.

[17] Telles S, Naveen KV (2008). Voluntary breath regulation in yoga: its relevance and physiological effects. *Biofeedback*.

[18] Laude D, Elghozi JL, Girard A (2004). Comparison of various techniques used to estimate spontaneous baroreflex sensitivity (the EuroBaVar study). *The American Journal of Physiology*.

[19] Bernardi L, de Barbieri G, Rosengård-Bärlund M (2010). New method to measure and improve consistency of baroreflex sensitivity values. *Clinical Autonomic Research*.

[20] Bertinieri G, di Rienzo M, Cavallazzi A (1985). A new approach to analysis of the arterial baroreflex. *Journal of Hypertension*.

[21] Pagani M, Somers V, Furlan R (1988). Changes in autonomic regulation induced by physical training in mild hypertension. *Hypertension*.

[22] Pinna GD, Maestri R (2001). Reliability of transfer function estimates in cardiovascular variability analysis. *Medical and Biological Engineering and Computing*.

[23] Tobin MJ, Jenouri G, Lind B (1983). Validation of respiratory inductive plethysmography in patients with pulmonary disease. *Chest*.

[24] Askanazi J, Silverberg PA, Foster RJ (1980). Effects of respiratory apparatus on breathing pattern. *Journal of Applied Physiology Respiratory Environmental and Exercise Physiology*.

[25] Bernardi L, Rosengård-Bärlund M, Sandelin A (2011). Short-term oxygen administration restores blunted baroreflex sensitivity in patients with type 1 diabetes. *Diabetologia*.

[26] van den Aardweg JG, Karemaker JM (2002). Influence of chemoreflexes on respiratory variability in healthy subjects. *The American Journal of Respiratory and Critical Care Medicine*.

[27] Bruning JL, Kintz BL (1968). *Computational Handbook of Statistics*.

[28] Waring WS, Thomson AJ, Adwani SH (2003). Cardiovascular effects of acute oxygen administration in healthy adults. *Journal of Cardiovascular Pharmacology*.

[29] Bernardi L, Passino C, Wilmerding V (2001). Breathing patterns and cardiovascular autonomic modulation during hypoxia induced by simulated altitude. *Journal of Hypertension*.

[30] Buda AJ, Pinsky MR, Ingels NB (1979). Effect of intrathoracic pressure on left ventricular performance. *New England Journal of Medicine*.

[31] Jerath R, Edry JW, Barnes VA, Jerath V (2006). Physiology of long pranayamic breathing: Neural respiratory elements may provide a mechanism that explains how slow deep breathing shifts the autonomic nervous system. *Medical Hypotheses*.

[32] Calabrese P, Dinh TP, Eberhard A, Bachy JP, Benchetrit G (1998). Effects of resistive loading on the pattern of breathing. *Respiration Physiology*.

[33] Spicuzza L, Gabutti A, Porta C, Montano N, Bernardi L (2000). Yoga and chemoreflex response to hypoxia and hypercapnia. *The Lancet*.

[34] Stanescu DC, Nemery B, Veriter C, Maréchal C (1981). Pattern of breathing and ventilatory response to CO_2_ in subjects practicing hatha-yoga. *Journal of Applied Physiology*.

[35] Bowman AJ, Clayton RH, Murray A, Reed JW, Feisal Subhan MF, Ford GA (1997). Baroreflex function in sedentary and endurance-trained elderly people. *Age and Ageing*.

